# Convergent evolution of elaborate nests as structural defences in birds

**DOI:** 10.1098/rspb.2022.1734

**Published:** 2022-12-21

**Authors:** Sally E. Street, Robert Jaques, Thilina N. De Silva

**Affiliations:** ^1^ Department of Anthropology, Durham University, South Rd, Durham DH1 3LE, UK; ^2^ British Trust for Ornithology, The Nunnery, Thetford IP24 2LP, UK; ^3^ Department of Ecology and Evolutionary Biology, Princeton University, 106A Guyot Ln, Princeton, NJ 08544, USA; ^4^ Department of Ecology and Evolutionary Biology and Biodiversity Institute, University of Kansas, 1345 Jayhawk Blvd., Lawrence, KS 66045, USA

**Keywords:** nest-building, weaverbirds, icterids, life history, phylogenetic comparative methods

## Abstract

The pendent nests of some weaverbird and icterid species are among the most complex structures built by any animal, but why they have evolved remains to be explained. The precarious attachments and extended entrance tunnels characteristic of these nests are widely speculated to act as structural defences against invasion by nest predators, particularly tree-climbing snakes, but this hypothesis has yet to be systematically tested. We use phylogenetic comparative methods to investigate the relationship between nest structure and developmental period length, a proxy for offspring mortality, in weaverbirds (Ploceidae) and icterids (Icteridae), two bird families in which highly elaborate pendent nests have independently evolved. We find that more elaborate nests, particularly those with entrance tunnels, are associated with longer developmental periods in both families. This finding is robust to potentially confounding effects of body mass, phylogenetic relationships, nest location and latitude. Our results are consistent with the hypothesis that elaborate nest structures in birds can function as structural defences, resulting in lower offspring mortality and slower development. More generally, our findings suggest that constructing complex, protective structures may buffer against environmental hazards, reducing extrinsic mortality and contributing to the evolution of slower life histories in diverse animal lineages, even humans.

## 1. Introduction

The structural complexity of birds' nests varies enormously across species, from roughly constructed stick platforms to neatly woven cups and domes [1–4]. The reasons that some bird species have evolved to build more complex nests than others, however, remain poorly understood due to a surprising historical lack of research interest in the evolution of nest-building [5]. Perhaps the most complex of all birds' nests are ‘pendent’ designs—enclosed domes dangling precariously from substrates above, resembling hanging-baskets [1–4]. Pendent nests are built by members of several passerine families, but the most elaborate examples are found among the weaverbirds (Ploceidae) and icterids (Icteridae) [1,4]. To build these nests, birds must knot, stitch and weave together hundreds of strips of nesting material [4,6], requiring a significant amount of physical effort, manipulative skill and trial-and-error learning [6–9]. Such nests, therefore, presumably confer substantial fitness benefits to compensate for the costs of their construction [3]. The primary advantage of pendent nests is widely assumed to be protection from arboreal predators, particularly snakes [2,4]. Anecdotal evidence suggests that snakes struggle to access nests suspended below slim branches [9], and that entrance tunnels hinder easy access by brood parasites [10,11]. While intuitive, this hypothesis is so far based largely on observational accounts and has yet to be investigated systematically in a phylogenetic comparative analysis.

**Figure 1.**
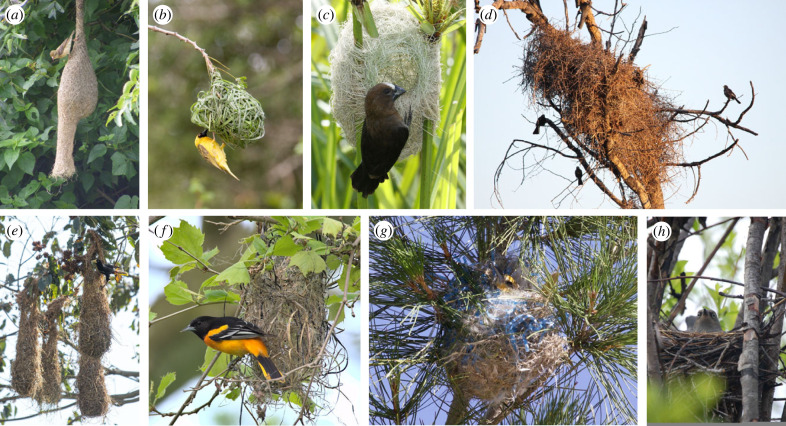
Examples illustrating the diversity of nest designs in weaverbirds (top row) and icterids (bottom row). (*a*) Baya weaverbird (*Ploceus philippinus*), (*b*) Southern masked weaver (*Ploceus velatus*), (*c*) Thick-billed weaver (*Amblyospiza albifrons*), (*d*) Red-billed buffalo weaver (*Bubalornis niger*), (*e*) Crested oropendola (*Psarocolius decumanus*), (*f*) Baltimore oriole (*Icterus galbula*), (*g*) Bullock's oriole (Icterus bullocki), (*h*) Rusty blackbird (*Euphagus carolinus*). Image credits: (*a*) Dr Raju Kasambe – CC BY-SA 3.0, https://commons.wikimedia.org/w/index.php?curid=19828748, (*b*) Chris Eason – CC BY 2.0, https://commons.wikimedia.org/w/index.php?curid=4127816, (*c*) Derek Keats – CC BY 2.0, https://commons.wikimedia.org/w/index.php?curid=45087290, (*d*) Derek Keats – CC BY 2.0, https://www.flickr.com/photos/dkeats/6041784294, (*e*) Daniel Ocampo Rincón – reproduced with permission, (*f*) Andrew Weitzel – CC BY-SA 2.0, https://commons.wikimedia.org/w/index.php?curid=105254878, (*g*) HarmonyonPlanetEarth – CC BY 2.0, https://commons.wikimedia.org/w/index.php?curid=45578683, (*h*) Robin Corcoran, USFWS – CC0, https://pixnio.com/fauna-animals/birds/blackbirds-pictures/female-rusty-blackbird-euphagus-carolinus-on-nest. All images have been edited only by cropping and re-sizing. (Online version in colour.)

Nest complexity varies considerably within the weaverbird and icterid families, making them ideal groups for testing hypotheses about the evolution of elaborate nest structures (figure 1). In weaverbirds, nests range from roughly constructed, bulky masses firmly sited on thick branches to neatly woven globes dangling precariously below slim vegetation, some with entrance tunnels up to a metre long [9,12]. In the icterids, nest complexity varies from typical songbird cups to suspended pouches and elongated purse-shaped nests, while some do not build nests at all, instead exploiting those built by other species [13]. Despite their independent evolution, pendent nests in the two families are highly similar in their design and construction: oropendolas and caciques use similarly intricate weaving methods, including some of the same stitches (e.g. half-hitches, loops and spiral binding) as do some of the weaverbirds [4,8]. Such striking convergence strongly suggests common selection pressures; threats of attack by snakes and brood parasites are particularly likely candidates given that they are a significant source of offspring mortality in both families [10–15].

Here, we conduct the first systematic test of the hypothesis that elaborate pendent nests in birds have evolved as structural defences, in a phylogenetic comparative study of weaverbirds (Ploceidae) and icterids (Icteridae). If elaborate structural features of pendent nests provide protection from nest invasion by arboreal predators or brood parasites, then species building more protected nests, i.e. those with tunnels and/or with more precarious attachments, should show evidence of reduced offspring mortality compared with species that build less protected nests. The evolution of species' life histories is strongly shaped by extrinsic mortality risks [16], and therefore life-history traits can be used as proxies for evolutionary responses to predation in comparative analyses (as in e.g. [17,18]). The length of time that developing offspring spend in the nest is of particular relevance to the present study. Theoretical models suggest that selection should favour rapid maturation where offspring are raised in exposed locations, while offspring raised in protected nests can afford to develop more slowly due to relaxed predation pressure [19]. In support of this assumption, multiple comparative analyses across diverse avian assemblages have shown that offspring developmental periods are shorter in species with higher rates of nest predation (e.g. [20–24]). While less widely investigated, higher rates of brood parasitism have also been shown to be associated with shorter developmental periods in birds [25]. We test predictions by examining the effects of nest design on species' developmental period length (incubation periods, nestling periods and their combined duration) using phylogenetic comparative analyses, accounting for potentially confounding effects of body mass, nest location and breeding latitude.

## 2. Methods

### (a) Data collection

We obtained data on nest design, life-history traits, body mass and latitude in weaverbird and icterid species from multiple secondary sources. We resolved mis-matches between species' names in different datasets and the phylogenies where possible by referring to the latest BirdLife International taxonomy [26]. We classified variation in nest design primarily based on descriptions and images of nests from Birds of the World Online [12,13]. We also obtained photographs of nests from the Natural History Museum at Tring, both via the NHM Data Portal [27] and from an in-person visit. After finding that information on weaverbird nests in Birds of the World Online was less comprehensive than for icterids, we consulted two additional sources for descriptions and images of weaverbird nests: PHOWN, a citizen science project collating photographs of weaverbird nests [28] and a comparative study of weaverbird nests conducted prior to the development of modern phylogenetic methods [9]. Where information conflicted between sources, we generally prioritized photographs over textual descriptions, taking into account image quality. Where there was insufficient information to classify species' nest designs (i.e. vague textual descriptions, poor quality images or no information at all), we conducted further targeted Google Image and Google Scholar searches (using the search string ‘[binomial] OR [common name] AND nest’) for further information. If we could find no reliable further information from these targeted searches, we excluded species from the analyses.

We classified weaverbird nests based on two separate design features: the presence of entrance tunnels, and the type of attachment (electronic supplementary material, figure S1). We considered nests to have tunnels if an external, tube-shaped extended entrance was clearly present, of any length. We did not count short extensions to the upper side of the nest entrance only (often referred to as ‘porches’ or ‘lips’) as tunnels. We treated nest attachment as a categorical variable with three levels: ‘supported’, ‘suspended’ or ‘pendulous’, increasing in precarity of attachment and therefore presumed difficulty of access by invaders. We classified as ‘supported’ nests those that are attached from the underside to a branch, built on the ground or firmly attached on two sides between vertical supports rising up from the ground. We classified as ‘suspended’ nests attached at the top or side(s), so that the bulk of the nest lies below the substrate, while we classified ‘pendulous’ nests as those hanging from the substrate above by a single point of attachment. For icterids, we treated nest design as a single three-level factor, ordered by precariousness of attachment (supported < suspended < pendulous, electronic supplementary material, figure S1). We classified as ‘supported’ icterid nests that are firmly attached to vegetation or other substrates from below (including nests built on the ground and inside cavities). We classified as ‘suspended’ bag-shaped pouches attached at the rim or by multiple ‘straps’, such that the bulk of the nest hangs below the substrate, while more elongated, purse-shaped nests as ‘pendulous’. ‘Pendulous’ nests in icterids have entrances at the top rather than the bottom as in weaverbirds (figure 1*e*, electronic supplementary material, figure S1), creating an upward-facing entrance tunnel. Therefore, we could not classify tunnels as a design feature separate from attachment type in the icterids as we did for the weaverbirds. We excluded icterid species that do not build their own nests, including brood-parasitic cowbirds (*n* = 5) and troupials reliant on the nests of other species (*n* = 2). Where there was intraspecific variation in nest design, we considered a feature to be present if it occurs within the typical range of nest designs for the species. For example, we considered tunnels present in species that build nests both with and without tunnels, and pendulous nests present in species that build both pendulous and suspended nests. Where available, we also recorded the maximum recorded entrance tunnel length for tunnel-building weaverbirds, and maximum recorded nest length (both in cm) in suspended or pendulous nest-building icterids.

Nest location may play at least as important a role as nest structure in protecting developing offspring from potential nest invaders [3]. Weaverbirds and icterids often build their nests in locations inaccessible to most terrestrial predators, attached precariously to the tips of slim branches high off the ground [9,12,13]. Building nests in thorny vegetation, over water, in large breeding colonies and/or close to the nests of aggressive stinging insects or predatory birds may also provide effective deterrents to a wide range of potential predators, regardless of nest structure [9]. An observational study of baya weaverbirds in India found that characteristics of nest location were in fact more strongly associated with fledging success than were those of nest structure [10]. Therefore, it was important to consider both aspects of nest structure and location in our analyses, particularly as they may be confounded with one another if elaborate nests tend to be built in more protected locations. Along with nest structure data, we collected data on potentially protective features of nest location including nest height, colonial nesting, nesting in thorny vegetation, nesting over water, nesting in association with raptors and nesting in association with stinging insects from Birds of the World Online [12,13]. Nest height was given in metres above the ground or water. Where ranges or multiple values were provided for nest height, we took the median of the minimum and maximum provided, otherwise we used single values. We treated ground-nesting as nesting at a height of zero metres. We counted as colonial nesters species that breed in colonies, regardless of colony size, and including those described as ‘loosely’ or ‘semi’ colonial, as well as those that nest both in colonies and solitarily. We did not count as colonial, however, species with uncertain descriptions of coloniality such as ‘appears to be colonial’ or ‘presumably colonial’. We classified species as nesting in thorny vegetation, over water, in association with stinging insects (e.g. ants, wasps, bees or hornets) and in association with raptors if there was at least one clear description or image of each relevant behaviour. We could not analyse the potential role of nesting associations with raptors in developmental period length as it was reported only in a very small number of species in our samples (*n* = 6 in weaverbirds, 0 in icterids). Additionally, we found an insufficient number of icterid species nesting in association with stinging insects (*n* = 3) to allow for statistical analysis.

We obtained data on incubation period duration (days), nestling period duration (days) and adult body mass (grams) primarily from Birds of the World Online [12,13]. We used female body mass where available, otherwise we used male body mass or body mass of unknown sex. We included data from captive populations to increase sample size, though preferentially selected estimates from the wild when both data from wild and captive populations were available. Where life-history traits were reported as ranges or multiple values, we took the median of the minimum and maximum estimates, otherwise we used single values. Because we found that we had nest data for more species than we had life-history data, we performed further targeted literature searches for additional life-history data to increase sample sizes. Here, we checked two large comparative avian life-history databases [29,30] and performed a Google Scholar search for each individual species’ binomial and common names with relevant life-history keywords (using the search string [‘binomial OR common name] AND incubation OR nestling OR fledging’). As a result, we obtained life-history data for an additional 6 weaverbirds and 7 icterid species. A complete list of sources of additional life-history data is available in electronic supplementary material, table S1.

Finally, we obtained data on species' breeding latitudes in order to control for a potentially confounding effect of latitudinal gradients in our analyses. Bird species breeding in tropical regions closer to the equator tend to have slower life histories, including longer offspring developmental periods [31], and enclosed nests are more common in tropical and Southern Hemisphere regions [32]. Therefore, effects of latitude on both offspring development and nest design could result in a spurious association between the latter two variables if not controlled for. We downloaded species distribution maps for all weaverbird and icterid species from the IUCN Red List [33], and transformed them to presence-absence matrices with a grid cell resolution of 0.1°, counting a species as present in a grid cell if its range covered at least 10% of the cell. One species, the Montserrat oriole (*Icterus oberi*) had a range so small that its presence was not registered in any grid cells, and so we instead manually input the latitudinal midpoint for the island of Monserrat as the breeding latitude for this species. We discarded records of uncertain presence (i.e. excluding presence codes 2=‘probably extant’, 3=‘possibly extant’ and 6=‘presence uncertain’), limited records only to those from species' native ranges (i.e. including only origin codes 1=‘native’ or 2=‘reintroduced’) and discarded records from the non-breeding range (i.e. including only seasonality codes 1=‘resident’ or 2=‘breeding’). We then extracted the latitude of the polygon centroid of the presence-absence matrices to represent the breeding latitude of each species. We transformed latitude to absolute distance from the equator (in degrees) so that increasing values indicate greater distances from the equator in either hemisphere.

After matching species between the datasets and phylogenies, 56 weaverbird and 48 icterid species remained with complete data on nest design and developmental periods. Of these, 15 weaverbird and 17 icterid species also had data on tunnel or nest length, respectively.

### (b) Data analysis

We ran all analyses in R (version 3.6.3) [34], using functions from the *caper* [35], *ape* [36], *phangorn* [37], *phytools* [38] and *letsR* [39] packages. To investigate whether developmental periods are longer in species with more protected nest designs, we fitted regression models in which either incubation period length, nestling period length or total developmental period length (summed incubation and nestling period) was the outcome variable, with nesting variables, body mass and/or latitude as predictors. We included body mass as a predictor in all models to control for allometric scaling of developmental periods with body size [30]. We use *p*-values to estimate the probability of the observed effects under the null hypotheses of regression coefficients of zero. We do not, however, specify any arbitrary thresholds for ‘statistical significance’ in advance since *p*-values are continuous quantities [40]. We log-10-transformed all continuous variables prior to analysis as they were generally positively skewed (apart from latitude, which was not normalized by log-transformation). For all analyses, we examined standard regression diagnostic plots and found no concerning patterns.

We used phylogenetic generalized least squares (PGLS) regression to account for the non-independence of species data caused by phylogenetic relationships [41,42]. PGLS analyses adjust regression coefficients according to the degree of phylogenetic influence in model residuals, estimating phylogenetic signal using Pagel's *λ* [41,42]. *λ* varies from 0 to 1, where 0 indicates no phylogenetic signal and 1 the maximum possible signal, assuming a Brownian motion model in which the amount of phenotypic change is directly proportional to evolutionary time [43,44]. We obtained a near-species-level multi-locus phylogeny for the weaverbirds based on 4 mitochondrial markers and 4 nuclear introns [45,46], constructed using Bayesian inference in BEAST v. 1.82 [47], executed with BEAGLE [48] and summarized the tree block in the form of a maximum-clade credibility tree. For icterids, we used a complete phylogeny based on 2 mitochondrial genes, 4 nuclear genes and whole mitochondrial genomes for selected species, constructed using maximum-likelihood estimation [49]. Primarily for illustrative purposes, we also performed ancestral states reconstructions on nest tunnels in weaverbirds and nest attachment in icterids. We used maximum-likelihood methods to estimate transition rates, fitting a simple model in which all transition rates were assumed to be equal [36]. We also calculated phylogenetic signal for these nesting traits using the *D* statistic, a measure based on the sum of differences in binary traits between sister clades [50]. Lower *D*-values indicate more similarity in traits between sister clades and therefore higher phylogenetic signal. *D*-values are scaled so that 0 indicates traits are clustered in line with expectations based on an underlying Brownian motion of evolutionary change, while values of 1 are consistent with a trait randomly distributed across the tips of the phylogeny. Negative *D*-values are possible, indicating extreme phylogenetic clustering, as are *D*-values of greater than 1, indicating overdispersion. Since the *D* statistic is suitable only for binary traits, here we treat nest attachment in icterids as binary where 0 = supported and 1 = suspended or pendulous nests.



## 3. Results

**Figure 2.**
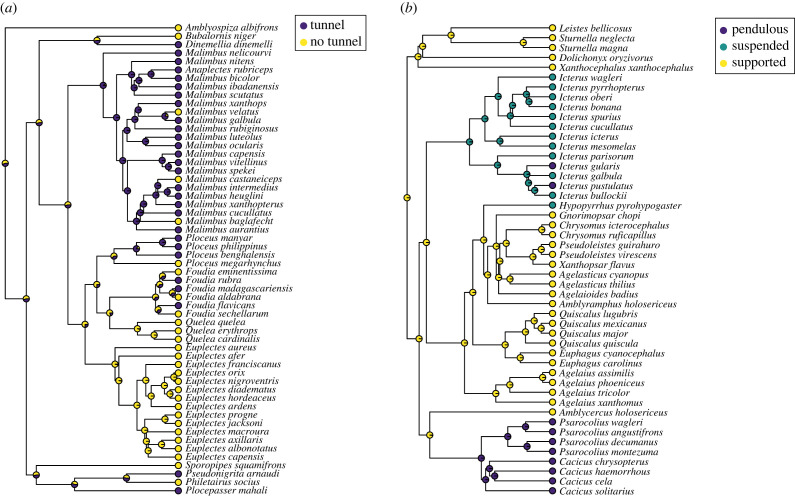
Nest characteristics mapped onto phylogenies for (*a*) weaverbirds and (*b*) icterids. Tip labels show observed nest classifications among extant species, while node labels illustrate the results of the ancestral states reconstructions, with shaded areas of the pie charts indicating the estimated probability of each state at each node. Both traits exhibit phylogenetic signal, with stronger phylogenetic clustering in icterid nest types (*D* = −1.39, *n* = 48) compared with tunnels in weaverbirds (*D* = −0.08, *n* = 56). (Online version in colour.)

Figure 2 illustrates variation in nest design across the weaverbird and icterid phylogenies and displays results of ancestral states reconstructions.

### (a) Weaverbirds

**Figure 3.**
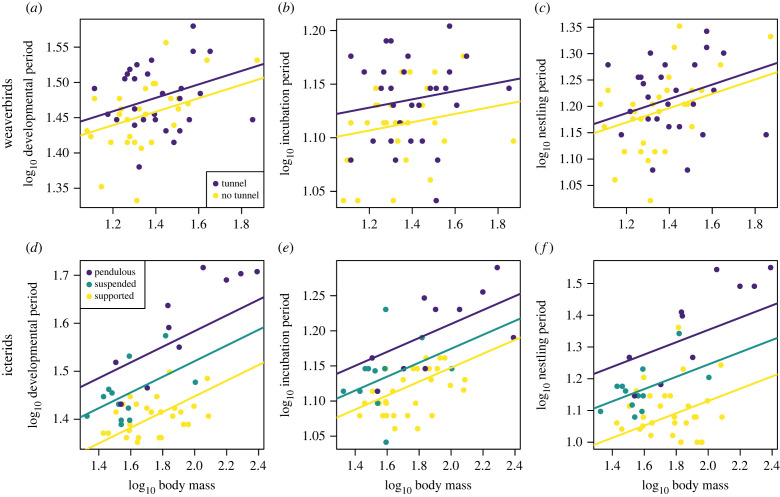
Scatterplots illustrating relationships between (*a*) nest tunnels and total developmental durations in weaverbirds, (*b*) nest tunnels and incubation periods in weaverbirds, (*c*) nest tunnels and nestling periods in weaverbirds, (*d*) nest type and total developmental durations in icterids, (*e*) nest type and incubation periods in icterids and (*f*) nest type and nestling periods in icterids. Fit lines are based on PGLS model coefficients. (Online version in colour.)

Weaverbird species building nests with entrance tunnels have offspring with slightly longer combined developmental periods than those building nests without entrance tunnels (figure 3*a*; table 1). Predicted values based on model coefficients suggest that building a nest with a tunnel is associated with an additional ~1.3 days from laying to fledging age, for a weaverbird species of average body mass. When separating developmental periods into incubation and nestling periods, we find that tunnels are associated with relatively longer incubation periods rather than nestling periods (table 1; figure 3*b,c*). Developmental periods differ little between weaverbird species building nests with pendulous, suspended or supported attachments, although pendulous nest-building species do have slightly longer developmental periods (incubation in particular) than suspended or supported nest-builders (table 2). Tunnel length and developmental period length appear not to be positively correlated, although statistical power is very low due to the small sample size for species with tunnel length data (*n* = 15; electronic supplementary material, figure S2*a–c*; table 3). In weaverbirds, nest height above the ground is not related to developmental period length (electronic supplementary material, table S2), and nesting in protected locations is generally not associated with longer developmental periods (electronic supplementary material, tables S3–S7), apart from longer incubation periods in species nesting over water (electronic supplementary material, table S5). Positive, though weaker, effects of both tunnels and nesting over water remain when both are included as predictors in the same model (electronic supplementary material, table S6). Developmental period length is not strongly associated with breeding latitude in weaverbirds, and relationships between nest tunnels and developmental periods remain when breeding latitude is included as an additional co-variate in the models (electronic supplementary material, table S8).

**Table 1.** Full results from models predicting log10 developmental period length, log10 incubation period length and log10 nestling period length from tunnel presence and log10 body mass in weaverbirds. The reference level is nests lacking entrance tunnels.

dep. variable	ind. variable	estimate	s.e.	*T*-value	*p*-value	*n*	*R*^2^	*λ*
dev. period	tunnel	0.02	0.01	1.60	0.11	56	0.18	0.00
	body mass	0.10	0.04	2.61	0.01			
incubation period	tunnel	0.02	0.01	2.14	0.04	58	0.12	0.00
	body mass	0.04	0.03	1.31	0.20			
nestling period	tunnel	0.02	0.02	0.94	0.35	57	0.13	0.00
	body mass	0.14	0.06	2.43	0.02

**Table 2.** Full results from models predicting log10 developmental period length, log10 incubation period length and log10 nestling period length from attachment type and log10 body mass in weaverbirds. The reference level is supported nest attachments.

dep. variable	ind. variable	estimate	s.e.	*T*-value	*p*-value	*n*	*R*^2^	*λ*
dev. period	suspended	0.00	0.02	0.26	0.80	56	0.18	0.00
	pendulous	0.02	0.01	1.32	0.10			
	body mass	0.10	0.04	2.67	0.01			
incubation period	suspended	0.00	0.01	−0.45	0.65	58	0.12	0.00
	pendulous	0.02	0.01	1.74	0.09			
	body mass	0.05	0.03	1.60	0.12			
nestling period	suspended	0.02	0.02	0.78	0.44	57	0.15	0.00
	pendulous	0.03	0.02	1.47	0.15			
	body mass	0.13	0.06	2.30	0.03

**Table 3.** Full results from models predicting log10 developmental period length, log10 incubation period length and log10 nestling period length from log10 nest tunnel length and log10 body mass in weaverbirds.

dep. variable	ind. variable	estimate	s.e.	*T*-value	*p*-value	*n*	*R*^2^	*λ*
dev. period	tunnel length	0.03	0.03	0.86	0.41	15	0.06	0.00
	body mass	−0.01	0.12	−0.09	0.93			
incubation period	tunnel length	0.02	0.02	0.98	0.35	15	0.19	0.00
	body mass	0.10	0.08	1.22	0.25			
nestling period	tunnel length	0.03	0.05	0.62	0.55	15	0.05	0.00
	body mass	−0.10	0.17	−0.59	0.56			

While there is phylogenetic signal in weaverbird nest design (figure 2), most of the weaverbird PGLS analyses reported *λ* values of 0. Because PGLS estimates phylogenetic signal based on the distribution of model residuals, the signal of individual variables in the model is not necessarily expected to be concordant with estimates of *λ* for the model as a whole [42,51]. However, low signal in this case was unexpected given that developmental periods are generally strongly influenced by phylogeny in birds [30]. Low *λ* values in PGLS models have a range of possible explanations including both biological factors, such as highly labile traits [52] and methodological artefacts, particularly low statistical power [43]. In our case, the latter was unlikely as we find *λ* values greater than 0 in some weaverbird analyses (and in analyses of icterids, even when sample sizes were smaller), and *λ* values are still 0 or relatively low (≤0.50) when estimating phylogenetic signal in each life-history trait individually (electronic supplementary material, table S9). Fixing *λ* to the maximum value of 1 in the PGLS models substantially decreases model fit based on *R^2^* and AIC values suggesting that the assumption of maximum phylogenetic signal according to a Brownian motion model of evolutionary change is inappropriate in this case (electronic supplementary material, table S10). We investigated this issue further by estimating Pagel's *δ*, which allows the rate of evolution in the underlying model of phenotypic change to vary through time, along with Pagel's *λ* [44]. *δ* varies from 0 to 3, where values of less than 1 suggest faster evolutionary change earlier in the phylogeny, consistent with adaptive radiations, while higher values suggest faster change later in the phylogeny, suggestive of recent convergent adaptations [44]. We found high (>2.2) values of *δ* for all three life-history traits in weaverbirds (electronic supplementary material, table S11), consistent with recent, rapid evolutionary change in independent weaverbird lineages. Effects of nest tunnels on weaverbird developmental periods are unaffected by estimating both *δ* and *λ* simultaneously in PGLS models (electronic supplementary material, table S12).

### (b) Icterids

Icterid species building pendulous nests (which incorporate upward-facing tunnels) have longer developmental periods compared with those building suspended nests, who in turn have longer developmental periods than those building supported nests (figure 3*d*, table 4). Model predictions suggest that the offspring of pendulous nest-building species require an additional ~4.7 days to reach fledging age compared with suspended nest-builders, who in turn take again ~4.7 more days than supported nest builders, assuming an icterid species of average body mass. In contrast to the weaverbirds, nest type has a stronger effect on the length of nestling than incubation periods (figure 3*e*,*f*, table 4). Nest length is positively, although fairly weakly, correlated with developmental period length, particularly nestling period, controlling for body mass (electronic supplementary material, figure S2*d*–*f*, table 5). Developmental periods (including both incubation and nestling periods) increase with nest height off the ground in icterids (electronic supplementary material, table S13). However, effects of nest type on developmental period length remain when both nest type and nest height are included in the same model (electronic supplementary material, table S14). Similarly to weaverbirds, in icterids we find no evidence that nesting in protected locations is associated with longer developmental periods (electronic supplementary material, tables S15–S17). There is a strong latitudinal gradient in icterid developmental periods: species breeding closer to the equator have more slowly developing offspring (electronic supplementary material, table S18). However, effects of nest design on developmental periods remain even when controlling for the effect of latitude (electronic supplementary material, table S18).

**Table 4.** Full results from models predicting log10 developmental period length, log10 incubation period length and log10 nestling period length from nest type and log10 body mass in icterids. The reference level is supported nest types.

dep. variable	ind. variable	estimate	s.e.	*T*-value	*p*-value	*n*	*R*^2^	*λ*
dev. period	suspended	0.07	0.03	2.73	0.01	48	0.54	0.64
	pendulous	0.14	0.03	4.52	<0.01			
	body mass	0.16	0.04	4.60	<0.01			
incubation period	suspended	0.03	0.02	1.31	0.20	50	0.36	0.66
	pendulous	0.06	0.02	2.69	0.01			
	body mass	0.10	0.03	3.66	<0.01			
nestling period	suspended	0.11	0.04	2.76	<0.01	52	0.46	0.37
	pendulous	0.22	0.05	4.86	<0.01			
	body mass	0.19	0.06	3.18	<0.01

**Table 5.** Full results from models predicting log10 developmental period length, log10 incubation period length and log10 nestling period length from log10 nest length and log10 body mass in icterids.

dep. variable	ind. variable	estimate	s.e.	*T*-value	*p*-value	*n*	*R*^2^	*λ*
dev. period	nest length	0.06	0.03	1.89	0.08	17	0.56	1.00
	body mass	0.16	0.06	2.96	0.01			
incubation period	nest length	0.03	0.03	0.89	0.39	17	0.34	0.45
	body mass	0.09	0.05	1.74	0.10			
nestling period	nest length	0.09	0.05	1.78	0.10	17	0.52	1.00
	body mass	0.22	0.08	2.67	0.02			

## 4. Discussion

We find that in both weaverbirds and icterids, species building more elaborate nests, particularly those with extended entrance tunnels, produce offspring with longer developmental periods. Since theoretical and comparative evidence shows that offspring develop more slowly under lower predation or brood parasitism pressure [16,19–25,53], these results are consistent with the hypothesis that nests with extended entrance tunnels limit the exposure of developing broods to nest invaders. The consistency of these findings is striking given that highly elaborate nests have evolved independently in the weaverbirds and icterids. We also find that in icterids at least, developmental period length is positively correlated with nest tunnel length, suggesting that longer tunnels are more effective at hindering access by nest invaders than shorter tunnels. We find some evidence that nesting in protected locations is also associated with longer developmental periods in these two families, including nesting over water in weaverbirds and nesting higher off the ground in icterids. However, effects of nest structure on developmental periods are not confounded by nest location. Our findings also cannot be explained by potentially confounding effects of latitudinal gradients on avian life histories [31] and nest design [32]. The low phylogenetic signal that we find in weaverbird developmental periods suggests that their evolution substantially deviates from expectations based on a Brownian motion-based, gradualistic model. Instead, the high values of Pagel's *δ* that we find are suggestive of recent, rapid convergent evolutionary changes in developmental periods across independent weaverbird lineages [44], consistent with the hypothesis that these traits have undergone strong selection following changes in nest morphology. Therefore, we provide the first comparative evidence in favour of the long-held hypothesis that elaborate pendent nests in birds have evolved as structural defences against nest invasion.

Multiple comparative analyses across diverse avian assemblages have shown that offspring developmental periods are negatively correlated with nest predation or (to a lesser extent) brood parasitism rates [20–25]. Therefore, offspring developmental periods are a reasonable proxy for offspring mortality risk from nest invaders in birds. Since life-history traits are shaped by extrinsic mortality risks over evolutionary timescales [16], offspring developmental periods are arguably more appropriate for testing hypotheses about the evolution of complex nest designs than present predation or parasitism rates, which are increasingly affected by anthropogenic environmental disturbances such as introduced species and climatic shifts. Nonetheless, we acknowledge that direct measures of age-related mortality risks would be helpful for investigating the protective function of elaborate nest structures in greater detail. Currently, there are few estimates of nest predation or parasitism rates in the weaverbirds and icterids available. We examined several recent, large-scale comparative analyses of daily nest predation rates (DPR) in birds and found data for only 5 of the weaverbird and 12 of the icterid species in our samples [20,21,54–59] (electronic supplementary material, tables S19 and S20). These samples are too small for formal statistical analysis, especially given that of those with DPR data, only 2 of the weaverbirds build nests with tunnels and 1 of the icterids builds a suspended nest. However, these data do suggest some patterns consistent with our hypothesis that elaborate nests reduce predation risk—among the icterids, the species with the lowest DPR is the Baltimore oriole (*Icterus galbula*) which is also the only species of this sample to build a suspended nest. Possible patterns for the weaverbirds are less clear, but we do tentatively note that the species with the highest DPR builds a non-suspended nest without a nest tunnel, the yellow bishop (*Euplectes capensis*). We hope that our study will inspire future compilations of nest predation data focused on the weaverbird and icterid families, allowing for more detailed insights into the behavioural ecology of these groups which contain many still-understudied species. It would also be interesting to investigate the possibility that complex nest structures reduce adult as well as juvenile mortality risk while offspring are in the nest, as both have been shown to be negatively correlated with developmental period length in birds [60], when suitable data become available.

While our results do not directly demonstrate which specific nest invaders elaborate nests have evolved in response to, they may suggest that brood parasites play a more important role than previously appreciated. Both precarious attachments and extended entrance tunnels should protect against attacks by arboreal snakes, yet in weaverbirds, where the two features are separable, we find that longer developmental periods are associated more strongly with tunnels than attachment type. Tunnels should make it physically more difficult for brood parasites to access nests quickly and avoid detection by hosts [10,11], while precarious nest attachments are of no obvious relevance to ease of access by brood parasites. The stronger effect of tunnels than attachment type on developmental periods in weaverbirds, therefore, is more consistent with protection against brood parasites than arboreal snakes. The finding that nest tunnels are associated with longer incubation periods rather than nestling periods in the weaverbirds is also consistent with an important role of nest tunnels in protection from brood parasites. A previous comparative analysis has shown that egg coloration is less variable in tunnel-building than non-tunnel-building *Ploceus* weavers, consistent with relaxed parasite pressure on species building structural defences [11]. Further, pendulous nests in the icterids are not obviously well-designed to prevent access by snakes since the nest entrance is at the top, allowing for relatively easy access from branches above. The potential role of brood parasitism in elaborate nest designs has so far been overlooked in comparison to snake predation, but remains a plausible explanation given the high risk of parasitism in many weaverbird and icterid species. The diederik cuckoo (*Chrysococcyx caprius*) alone targets at least 34 different host weaverbird species [61], with rates of parasitism as high as 50% in some populations (e.g. southern red bishops, *Euplectes orix* [14]). The vast majority of icterids are parasitized by at least one cowbird species [62], with rates of close to 100% parasitism sometimes reported (e.g. orchard orioles, *Icterus spurius* [15]). Brood parasitism, therefore, may exert significant selection pressures on nest design in weaverbird and icterids.

By contrast to nest structure, we find that nest location has generally little effect on offspring developmental periods across weaverbird and icterid species, other than nesting over water in weaverbirds and nesting high off the ground in icterids. This result is perhaps surprising given that many prior observational studies have found that multiple aspects of nest location, particularly nest height, affect exposure to predators [3]. Our results appear to conflict, for example, with a prior study of baya weaverbirds (*Ploceus philippinus*) which found that both nesting at greater heights and in thorny trees increased the probability of fledging success, while entrance tube length had no significant effect [10]. This study, however, seemed to capture only a limited amount of variation in entrance tube length among baya weavers: nests within the study population had entrance tubes up to only 14 cm long while they can reach as long as 90 cm in this species [10]. Baya weavers, further, may deviate from general patterns in the weaverbirds as they are not parasitized by cuckoos and may be affected more by rodent than snake predation [10]. A greater number of population-level studies on the role of nest structure and location in offspring survival across a wider diversity of weaverbird species would therefore be valuable for a more detailed understanding of how varying levels of predation and brood parasitism affect the design of elaborate nests both within and across species. Our findings also seem to conflict with those of a recent global-scale comparative analysis in birds which found that developmental durations increase with nest height, but not with more protected nest structures [30]. This study, however, focused on broad-scale comparisons between open and closed nest structures rather than on more elaborate features, such as tunnels, which are found only in a limited number of bird families. The weaverbirds and icterids likely deviate from these general patterns due to the exceptionally elaborate designs found in these families.

Birds' nest design is undoubtedly influenced by multiple selection pressures in addition to predation and brood parasitism, particularly climatic conditions [1–4]. However, while protection from heat, wind and rain could conceivably help explain why weaverbirds and icterids nests are enclosed and built from strong, tightly woven fabric [9], climate has no obvious connection to the construction of entrance tunnels in most species. In the large, communal nests of sociable weavers (*Philetarius socius*), more centrally located nesting chambers with longer internal entrance tunnels provide greater insulation from external temperature fluctuations than those nearer the edge of nests with shorter tunnels [63]. However, these nest structures are unusual among weaverbirds; such thermoregulatory benefits are perhaps less likely for the more typical, porous nest tunnels that hang down outside of the nest entrance, such as those of baya weavers (*Ploceus philippinus*, figure 1*a*). Elaborate nest designs in birds may also be shaped by mate choice in some species [3], a suggestion that appears compelling for weaverbirds given that unusually for birds, the most elaborate nests are typically built solely by males [9,12]. In some species, such as village weavers (*Ploceus cucullatus*), males appear to draw attention to the quality of their constructions by hanging upside down from nests during mating displays, and females seem to carefully inspect the neatness of their weaving before accepting the nest [64]. However, similar sexual selection pressures are unlikely to explain the convergence of elaborate nests in the weaverbirds and icterids since pendent nests are predominantly built by females rather than males in the latter [4,13]. The idea that sexual selection has shaped the design of pendent nests is yet to be investigated in a comparative analysis, although population-level studies have so far not supported it. In baya weavers, female choice is influenced primarily by nest location rather than nest structure [65], and while female village weavers prefer nests made with fresher material, their choices are primarily determined by male behaviour and mate quality [64,66]. Overall, therefore, while climate and sexual selection may generally be important drivers of nest design in birds, protection from predators and/or brood parasites appears the most plausible explanation for the evolution of elaborate nest structures in the weaverbirds and icterids.

In conclusion, our study suggests that highly elaborate pendent nests have evolved convergently in two bird families in response to similar threats to offspring survival from predators and/or brood parasites. Ours is the first comparative study to identify potential explanations for the evolution of such elaborate nests since John Crook's foundational study in the 1960s [9], taking advantage of the development of modern phylogenetic comparative methods, increasing availability of molecular phylogenies and compilation of large online comparative datasets. Our findings support the long-held, but until now untested, assumption that elaborate nest designs in weaverbirds and icterids function as structural defences, suggesting that pendent nests may have evolved independently in multiple passerine lineages in response to common threats from nest invaders. More broadly, our findings support the idea that by constructing protective structures, some animal species can exert greater control over their exposure to environmental hazards through behaviour, lowering extrinsic mortality risk and facilitating the evolution of slower life histories [67,68]. The ability to build protective structures for raising offspring may therefore help to explain why birds have such long lifespans relative to their body sizes, along with other key adaptations such as powered flight [17]. More broadly, construction of complex shelters may have contributed to extended life histories in a wider range of animal architects, including burrowing mammals [17] and potentially even our own species.

## Data Availability

All data and code used to produce the analyses reported in this study are available from the Dryad Digital Repository: https://doi.org/10.5061/dryad.ttdz08m0c [69]. Electronic supplementary material is available from Figshare [70].
